# Composable and executable scenarios for simulation-based testing of mobile robots

**DOI:** 10.3389/frobt.2024.1363281

**Published:** 2024-08-02

**Authors:** Argentina Ortega, Samuel Parra, Sven Schneider, Nico Hochgeschwender

**Affiliations:** ^1^ SECORO Group, Department of Computer Science, University of Bremen, Bremen, Germany; ^2^ Intelligent Software Systems Engineering Lab (ISSELab), Department of Computer Science, Ruhr University Bochum, Bochum, Germany; ^3^ Institute for AI and Autonomous Systems, Department of Computer Science, Hochschule Bonn-Rhein-Sieg, Sankt Augustin, Germany

**Keywords:** verification and validation, software testing, simulation-based testing, scenario-based testing, robot software engineering, model-based development, mobile robot, navigation

## Abstract

Few mobile robot developers already test their software on simulated robots in virtual environments or sceneries. However, the majority still shy away from simulation-based test campaigns because it remains challenging to specify and execute suitable testing *scenarios*, that is, models of the environment *and* the robots’ tasks. Through developer interviews, we identified that managing the enormous variability of testing scenarios is a major barrier to the application of simulation-based testing in robotics. Furthermore, traditional CAD or 3D-modelling tools such as SolidWorks, 3ds Max, or Blender are not suitable for specifying sceneries that vary significantly and serve different testing objectives. For some testing campaigns, it is required that the scenery replicates the dynamic (e.g., opening doors) and static features of real-world environments, whereas for others, simplified scenery is sufficient. Similarly, the task and mission specifications used for simulation-based testing range from simple point-to-point navigation tasks to more elaborate tasks that require advanced deliberation and decision-making. We propose the concept of *composable and executable scenarios* and associated tooling to support developers in specifying, reusing, and executing scenarios for the simulation-based testing of robotic systems. Our approach differs from traditional approaches in that it offers a means of creating scenarios that allow the addition of new semantics (e.g., dynamic elements such as doors or varying task specifications) to existing models without altering them. Thus, we can systematically construct richer scenarios that remain manageable. We evaluated our approach in a small simulation-based testing campaign, with scenarios defined around the navigation stack of a mobile robot. The scenarios gradually increased in complexity, composing new features into the scenery of previous scenarios. Our evaluation demonstrated how our approach can facilitate the reuse of models and revealed the presence of errors in the configuration of the publicly available navigation stack of our SUT, which had gone unnoticed despite its frequent use.

## 1 Introduction

The responsible deployment of autonomous mobile robots in everyday environments (e.g., warehouses, hospitals, and museums) relies on extensive testing to ensure that robots achieve their expected performance and can cope with failures to avoid safety risks during their operational lifetime. The two major types of testing–in simulations and the real world–have complementary properties. The former allows robots to be exposed to a wide range of situations early in the development cycle at a limited cost (Sotiropoulos et al., 2017; Timperley et al., 2018), whereas the latter offers more realistic situations and failure cases in later stages of the development cycle. Often, developers forego simulation-based testing, even if they are aware of its benefits, and expose their robots exclusively to the real world (Ortega et al., 2022). This often requires more time to set up than a simulator, and reduces coverage because it is difficult to change the real world, for example, by deliberately injecting failure-inducing situations. Both approaches can be employed for black- and white-box testing at various levels of abstraction (e.g., system vs. component tests).

In our previous study (Parra et al., 2023), we obtained a better understanding of why robot software engineers opt out of simulation-based testing by conducting in-depth interviews with 14 domain experts in the field of mobile robot navigation in indoor environments. One key insight we identified is that creating scenery or virtual environments in which simulated robots are deployed and tested remains challenging for developers. The use of traditional Computer Aided Design (CAD) and three-dimensional (3D) modelling tools is time-consuming because they require an additional skill set. To make simulation-based testing more attractive to developers, we designed and implemented two domain-specific languages (DSLs), namely, the FloorPlan DSL and the Variation DSL. We demonstrated how these DSLs enable developers to specify and automatically generate varying yet testable environments, and how testing robots in different simulated worlds overcomes the false sense of confidence (Hauer et al., 2020). Furthermore, our tooling helped discover real-world dormant bugs in the well-known ROS navigation stack (Parra et al., 2023).

Even though providing tool support for specifying testing scenery is a crucial element to make simulation-based testing of robot software more attractive, it is not sufficient. Additional models that express robot tasks and missions, robot capabilities, interactions among agents, and temporal evolution of actions and events are required to make simulation-based testing campaigns more realistic. In the field of autonomous driving, these models are known as *scenarios* (Tang et al., 2023). In the context of this study, we broadly define scenarios entailing both *mission-relevant* and *mission-plausible* information. On the one hand, by mission-relevant information we refer to, among others, the environment and its dynamics, time and events, objects (e.g., rooms) and subjects (e.g., human operators), and their potential behaviour. On the other hand, the mission-plausible information describes acceptance criteria that enable the verification and validation of the robotic requirements.

As we will show in this article, the interviews revealed that testing scenarios are characterized by a large amount of variability that results in varying, heterogeneous models expressing all too often implicitly in an ad-hoc way the robots’ environment and task, as well as the developers’ testing objectives, means to execute scenario models in simulations, and hints on how to collect and interpret test results. Therefore, simulation-based robot testing remains limited to carefully designed testing campaigns in which developers have control over a few scenario features and parameters, such as the type of robot task and the characteristics of the environment. Thus, reusing scenarios in the context of other testing campaigns is limited and a major barrier to achieving a higher level of test automation.

To improve this situation, we propose the concept of *composable and executable scenarios* and developed associated tooling to support robot software engineers in specifying, reusing, and executing scenarios in (semi-)automated simulation-based testing campaigns of robotic systems. To this end, we revisit and further analyse the corpus obtained by in-depth interviews conducted and briefly presented in our previous work (Parra et al., 2023), with the objective of deriving a domain model of scenario-based testing through simulation in robotics. As a result, we identified the common and variable parts of simulation-based testing and represented them in a feature model for scenarios of mobile robots. These features are selected to design or reuse the composable models needed for a particular scenario. Our composable modelling approach enables the addition of new semantics to existing scenarios, without altering them. This approach allows the development of new extensions and tools to support new use cases for the FloorPlan models previously introduced. To summarize, our contributions are:

• a domain model with common and variable features for simulation-based testing scenarios of mobile robots,

• a composable modelling approach to specify and execute scenarios,

• a dynamic-objects extension to the FloorPlan DSL that allows to model scenery objects and their locations using JSON-LD and facilitates the reuse and exploitation of environment models in simulation-based test design and semi-automated model-based scenario generation,

• three gazebo plugins that exploit the scenery information and integrate with the simulation to define the (initial) poses of objects (initial pose plugin), and actuate their joints on a time (dynamic joint plugin) or distance basis (distance-to-trigger plugin),

• a proof-of-concept tool to exploit scenery information and features from the FloorPlan DSL models to generate task specifications,

• and we demonstrate how one can use our approach to systematically run a simulation-based testing campaign with scenarios of varying complexity.

## 2 Domain analysis

To develop our composable and modelling approach, we perform a domain analysis using the corpus we obtained in (Parra et al., 2023). In this section, we describe the methodology we followed and the domain model we derived based on our insights.

### 2.1 Methodology

Semi-structured interviews were conducted (Hove and Anda, 2005), which involved interviews with specific questions to set the theme for the discussion, but allowed for exploration of the topic through open-ended questions. This allowed for a flexibly guided discussion. We designed a questionnaire that covered experts’ experience with software for mobile wheeled robots (specifically indoor navigation stacks for mapping, motion planning, and obstacle avoidance), their real-world challenges, and the challenges of simulation in the context of testing. An internal pre-study was conducted to improve the questionnaire. We then recruited participants for the study by reaching out to professionals in academia and the industry. A list of potential candidates was obtained from our professional network.

We conducted 14 interviews with a pool of experts with diverse academic and professional backgrounds, as well as multiple years of experience in the field. The table summarizing the interviewee’s demographics can be found in the Supplementary Material of this paper. The interviews were conducted through an online meeting, recorded, and transcribed into protocols, which were later separated into fragments. Interview participants signed a written informed consent and their participation was voluntary. All the interview data were anonymized by IDs, which only participants have and can use to withdraw their participation.

To analyse the fragments, we used qualitative coding (Saldaña, 2021), which consists of assigning one or multiple “codes” to the fragments^1^. For instance, the fragment “*One metric to measure map quality is to see how many tasks can be completed with it. How useful it is to solve certain kind of tasks.*” has the codes *Environment Representation* and *Performance*. We selected a list of codes before the start of the coding and allowed for expansion if necessary. We performed two rounds of coding: an initial round and a review in a second round. We used 37 themes to code the interviews. The distribution of references per individual code is available in the Supplementary Material of this paper. Once all the fragments were coded, patterns were identified in the data.

### 2.2 Domain model

**FIGURE 1:**
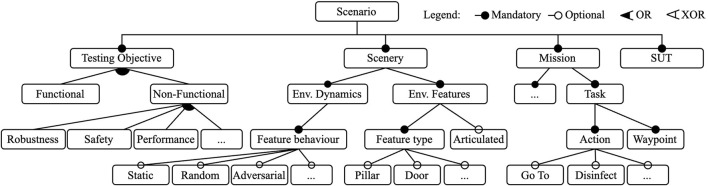
Abstract scenario feature model derived from the codes in participant interviews.

Based on the identified patterns, we derived a domain model for scenario-based testing in robotics in the form of a feature model, as illustrated in Figure 1. Here, we employed a standard feature model notation (Kang et al., 1990) to express the mandatory and optional features of the scenarios. Our scenario domain model is composed of four main features: the *System under Test (SUT)*, which is tested, assessed, and evaluated in the context of varying scenarios; the *testing objective* of the scenario; the *scenery*, which is a description of the environment or virtual world in which the SUT is embedded; and a specification of the *mission* that the SUT is expected to execute. In the following paragraphs, we explain the domain model by referencing some representative quotes from the interviews shown in Table 1 as excerpts E1-E10. Note that the abstract feature model in Figure 1 is not exhaustive; its abstraction levels were chosen to allow the addition of new features (e.g., planning and scheduling features under the mission feature) in future extensions.

**TABLE 1:** Representative interview excerpts and their relation to top-level features of our domain model.

Feature	ID	Excerpt
Testing Objective	E1	*I would measure [robustness] by trying challenging scenarios, maybe introducing different test. Create a simple environment for a test, such as a static environment, and make it more complex by adding dynamic obstacles*
	E2	*Another of my projects was regarding robot collisions, so a lot of tests also focused on that. The test were performed to optimize parameters and try to make the stack work*
	E3	*There is an impact, if there is a discrepancy between what you see in the real world and the map this will degrade the performance*
SUT	E4	*My goal was to integrate the platform and the navigation stack, so my tests had that objective*
Mission	E5	*We also deployed robots in industrial spaces, and there the setting was an industrial warehouse with many racks*
Scenery	E6	*We try to replicate the real environment, but is very limited. We have only a static world with the walls and objects that make the environment*
	E7	*Another challenge are dynamic obstacles, and understanding the environment. i.e., understanding that a piece of furniture is not fixed but also that it does not move often*
	E8	*Interacting with objects such as doors and chairs is also challenging*
	E9	*Lighting is one of the main issues if you wanna use VSLAM map, model of human agents are difficult in our standard simulator. I also wanted to model doors that open and close. It is interesting to simulate if the robot can get through certain doors*
	E10	*When dynamic come into play, i.e., everything that makes the map to change significantly, this can lead to localization and navigation failures*

The interviews revealed that the roles and activities of the developer influenced the type and scope of the tests they performed during the development process. Most interviewees considered themselves to be integrators and/or robot application developers in various fields, such as logistics or healthcare, where robots (cf. E4) perform missions characterized by navigation tasks and where an action is associated with one or more waypoints (e.g., the waypoints of racks to be visited in a logistic mission) (cf. E5).

Stakeholders mentioned a number of testing goals that influence their design decisions for their tests. Developers often build systems by composing readily available components (cf. E2), some of which are well-tested software packages developed and maintained by a third party such as an open-source community. Often, the components chosen for building the system are highly tailorable, which requires tuning parameters for an optimized performance (cf. E2). For integrators, interest in testing focuses on the capabilities and performance of the integrated system. These tests verified that all components were integrated correctly and validated the parameter values, and often involve multiple components and algorithms, instead of focusing on a single component. They are also more likely to require execution in a robotic simulator, and therefore, a scenery. However, other testing objectives such as safety and robustness, functional correctness, etc., are also present (cf. E1, E2, E3).

Generally, simulation is seen as a valuable tool, but it can be challenging to fully utilize it. The setup process for simulation execution can be a time-consuming task, meaning that smaller developer teams often opt to perform tests exclusively in the real world. One participant states, “*[using a simulator] depends on whether creating the simulation was going to add a long term benefit. In most cases, the answer was not. It required too much effort*”.

Creating or specifying the scenery, or environment model, for the simulation is often mentioned as one of the big challenges. Although simulating a scenario requires several types of models, such as robotic platform models, simulation capabilities for sensors and actuators, and a model of the environment (the scenery), the former two are often provided by their respective manufacturers, but the latter must often be created by application developers. We identified that scenery can be broadly divided into two main features, namely, the environment dynamics and the environmental features that are present. If the target environment contains particular features such as double doors, rails, or columns, it is useful to include them in the simulated environment to observe the behaviour of the system when it is exposed. (cf. E7) The simulation environment can also be application dependent. One participant stated, “*You need to describe the elements you want to be robust against. You do not want to describe all aspects.*” Modelling the 3D scenery for simulation is often the reason developers refrained from employing simulators. The experts see the modelling task as time-consuming, as one participant asserts “*The environment is very large and modelling is time-consuming*”. The effort necessary to model the environment depends on the scale and level of granularity that the test requires. The same participant states: “*Depending on the application, I would also like to see the models have either a lot of detail or be very simple*”. This refers to the levels of granularity, i.e., how much correspondence there is between the real world entity and its model (Hauer et al., 2020). Because modelling using traditional tools is time-consuming, and the 3D modelling tools have a steep learning curve, when developers create scenery models for simulation these tend to be low in granularity; i.e., they mostly consist of a set of walls.

The experts are also interested in re-creating environments for simulation. Two-thirds of the participants have tried to replicate the real world in a simulation. When real-world environments are re-created, most of the features of the environment were not modelled. The result is a scenery that consist of a set of walls that replicates the geometric shape of the original environment, with some cases adding objects such as furniture.

In summary, developers usually test their robots in scenery resembling static and primitive environmental features, such as walls and rooms (cf. E6) of the known and unknown target environment. These simple sceneries are incrementally enriched through additional and not necessarily dynamic features such as static obstacles (cf. E2) until the point of including dynamic elements, such as other agents, obstacles, and lighting conditions (cf. E9, E10), and actuated environmental features such as drawers, windows, and doors (cf. E8) to gain confidence in the tests. One can infer that developers of real-world robot applications would like to further exploit simulation-based testing of robotic systems, but that the current tools for specifying and executing actual test scenarios are limited. They do not allow the creation of scenery models in a flexible and incremental manner in which new concepts and associated semantics (e.g., a door and how it moves) can be added without altering the existing model.

## 3 Composable and executable scenarios

**FIGURE 2:**
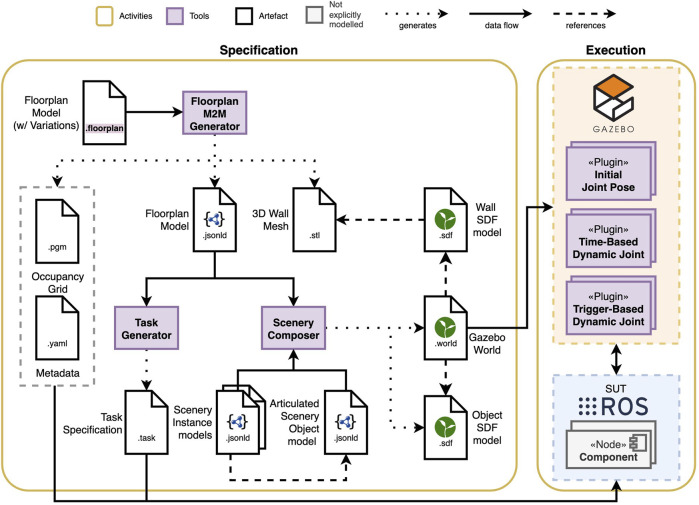
Composable and executable scenario pipeline showing the models, the tools discussed in this paper (purple) and the execution artefacts resulting from their composition and transformation.

One of the goals of our composable and executable scenario approach is to enable engineers in specifying, reusing and executing scenarios in simulation. Before specifying a scenario, examining the design space of the scenarios (cf. Figure 1) with the testing objective and the application requirements in mind results in the choice of the scenario features. Next, the corresponding models for those features must be specified (or potentially reused from other scenarios). Finally, these models are composed and transformed into software artefacts that can be used in simulation. The remainder of this section looks at these three main steps in more detail. Figure 2 shows an overview of the scenario specification and execution activities and tooling.

### 3.1 Scenario design

Let us start by examining the design dimensions of a scenario, and how the design decisions have an impact in the effectiveness and efficiency of the scenario.

The first design dimension to be determined should be the testing objective, as all the other design decisions for the scenario will depend on the objective and the scope of the test. There are numerous objectives that engineers can have in mind when designing a test, among others, examples include:

• Performance: Optimization of the configuration parameters for a particular behaviour in an exploratory way, identification of the effect of changes in the performance, or measurement of the efficiency of a given configuration.

• Robustness: Determination of the reaction or handling unexpected environmental changes, or calculating through experimentation the failure rates of the hardware or software components.

• Safety: Validation of conformance to internal or external standards, or identification of hazards and failures in the robot capabilities.

• Functional: Validation of the correctness of a component by validating that its performance is within the required or specified tolerances.

The task specification–*what* the SUT should do during a test–is one of the inputs that must be defined or adapted to support the test objective of the scenario and the scope of the SUT. Usually, the task for a fully-integrated system is determined by the application, but given the environmental complexity different variations of tasks, e.g., in scale or choice of locations, can be chosen to fit the SUT, and the scope and objective of the scenario. For instance, functional tests require tasks that are designed to be successfully completed in nominal conditions. Note that in unstructured environments, even for nominal conditions, larger scale or more complex tasks can reveal unexpected behaviours, due to the increasing amount of time the robot interacts with its surroundings. For other types of tests like safety, engineers can specify tasks informed by the test objective, e.g., choosing actions or constraints that could produce a failure. In short, the task specification is a test input that describes the workload the robot is expected to execute.

The scenery features include the features to be modelled in the floor plan, including the types of objects the robot interacts with, and the behaviour of those features, particularly if they are dynamic. The choices depend on the objective of the test (e.g., narrow hallways, moving obstacles), the SUT (e.g., minimum width of doorways for it to pass through). Functional tests require scenery that represents expected operating conditions, while sceneries that are used or designed for robustness tests must include features that represent invalid inputs or stressful conditions, such as dynamic obstacles. For testing the conformance to safety features, the scenery design should focus on including features that introduce hazards, increasing the risk of a critical failure. Consider the following examples of sceneries for different types of tests:

• Functional navigation tests: To assess if the robot is able to complete navigation tasks of varying complexity, validate if the robot is able to reach the target poses. A passing test means the robot reaches all the waypoints in its task. The complexity of the mission is determined by several factors: how many waypoints must be visited, the distance between waypoints, and the reachability from one waypoint to another. The distance between waypoints can be chosen from an existing scenery, or a new scenery can be created to test in larger environments. The reachability is constrained by the geometry of the space and by the pose of obstacles. The ideal simulation scenery for testing localization components includes many static features in the floor plan, but a reduction of these features can also increase the complexity.

• Robustness testing for obstacle avoidance: To validate how well the local planner adapts to sudden changes in the scenery, the robot can be tasked to perform navigation tasks of varying complexity in scenery where there are dynamic changes. The changes should be sufficient to trigger a re-planning of the planned path by the local planner, but not enough to worsen the localization performance. For this objective, it is sufficient that the changes occur at random times, where the complexity increases with the frequency and number of changes. This type of simulation scenery could also be used to perform a functional test of the trajectory planning component.

• Safety conformance by negative testing: One way to test the conformance to safety and functional requirements is to design a test case where the robot is expected to fail. Rather than random changes to the scenery, the changes can be adversarial to the robot, where especial conditions trigger changes in the simulation scenery. For instance, when the robot is less than 1 m away from the door, the door closes.

The test oracle–the mechanism to compare the expected result of a test with the observed output–is directly related to all the scenario features. Although they are usually derived from or influenced by the application requirements, they must be defined taking into account the objective of the test (e.g., what metrics to observe), the task (e.g., waypoints, specified tolerances or constraints), SUT (e.g., configuration) and scenery (e.g., free space, objects, obstacles). For example, the specified tolerance for the performance of a component can be the difference between a passed and failed test.

### 3.2 Scenario specification

A scenario specification is a composition of multiple models, with each individual model targeting a different dimension of the scenario. To form a complete specification of the scenario, we use composable models. A model is “composable” if the entities of the model can refer to each other via identifiers. New entities from a new model are composed by referencing the entities in the existing models. A model can now be a domain-specific artefact, that with composition can create a full specification. In previous work (Schneider et al., 2023), the use of JSON-LD as a representation for composable models was introduced, as well as many metamodels that are used in the scenario specifications presented in this work.

Composabiliy enables a modular approach for the re-use of models in multiple specifications. This is used in our approach as a way to systematically and gradually introduce complexity, and allows the creation of scenarios that are more challenging based on simpler scenarios without modifying the existing models. For instance, a scenario can start with a static scenery that just contains walls, and a new scenario reuses the floorplan model and composes a new simulation scenery with obstacles in the environment. The next scenario reuses these specifications and composes some dynamic behaviour to the obstacles, and so forth.

The floor plan model is the starting point of the scenery specification. We make a distinction between “user-facing” or “front-end” models and “machine-readable” or “back-end” models. Models written using DSLs are “front-end” models, as they are written using syntax and semantics meant for human understanding. On the other hand, the composable models are meant to be created and understood by computers. While it is possible to create these models by hand, it is complex and error-prone. A better approach is to transform the “front-end” models into “back-end” models.

The FloorPlan DSL, introduced in our previous work (Parra et al., 2023), is the base of the front-end environment specification. It enables developers to describe concrete indoor environments using a pseudo-code-like representation. The language is implemented with TextX (Dejanović et al., 2017), a Python-based language workbench for defining the metamodel and language syntax. The language is declarative and designed to be easy to understand. Using keywords such as Space, Column, or Entryway followed by an identifier, common elements of an indoor environment can be specified and referred to.

To compose objects, such as doors with hinges or elevator doors, into the scenery, their models must be specified in a composable way. We do so based on the kinematic chain metamodel described in previous work (Schneider et al., 2023) and represent them also in JSON-LD (as there is no front-end language currently available). Two types of scenery models are needed to represent an object: An object model describes their geometry and instance models that specifies the pose of its articulated joint using a selection of frames of reference.

### 3.3 Scenario execution

Executing the scenario involves the composition and transformation of the models into software artefacts for their execution in simulation. The execution of an indoor scenario requires multiple software artefacts: a simulation scenery (3D mesh) representing the walls of the environment for the simulator, an occupancy grid map representative of the environment for the navigation stack, and a task to complete in a format supported by the system.

Although the majority of the artefacts generated by the tools are simulator-independent, engineers will also need simulator-specific artefacts to run the tests; our current version of the tooling supports the generation of the artefacts required by the Robot Operating System (ROS) and the Gazebo simulator^2^. Previously, when the FloorPlan M2M Generator was executed, it used the manually-specified FloorPlan model to generate the occupancy grid maps and the 3D meshes that would be referred to by manually-specified Gazebo models and worlds. These sceneries could only represent static environments.

**FIGURE 3:**
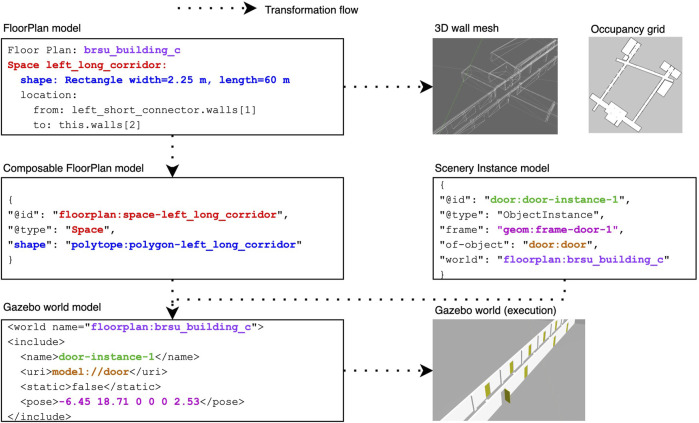
Scenery models are composed using their identifiers and then transformed into execution artefacts.

In this paper, we introduce an extension to the FloorPlan M2M Generator that generates a Composable FloorPlan model (represented in JSON-LD) to enable its composition with other scenery models. Now, it also generates the Composable FloorPlan models, where each entity has an identifier that other entities can refer to. The transformation and composition process, illustrated in Figure 3, links all the entities from the different models in a singular graph though their identifiers. Using this graph, we can make queries about environmental concepts and features, and generate artefacts or new models. The transformation and composition engine is implemented by using rdflib^3^ to query the singular graph, and, similar to textX generators, filling out a jinja template^4^ for the corresponding artefact. In our case, it allows us to model and compose objects into the scenery, and to define their dynamic behaviour.

Scenery composition refers to the composition of the static scenery models with the dynamic scenery objects to generate the simulation scenery. We developed the Scenery Composer tool to add articulated dynamic objects to the static simulation scenery from the FloorPlan models. The composable models enable the specification of the objects and their location in the environment, and the Scenery Composer tool creates a single scenery model that refers to all the different models together. The format of this model will depend on the simulator. In the case of Gazebo, this format is known as the Simulation Description Format (SDF)^5^, and referred to as the “world” file. At the time of writing, the Scenery Composer targets only Gazebo, and generates all the required models in SDF.

At runtime, three Gazebo plugins are responsible for the behaviour of the dynamic scenery objects. The three plugins require that the scenery object is articulated, i.e., has at least a revolute or prismatic joint. All plugins are able to set a joint pose, but differ when and how the changes occur. The Initial Joint Pose plugin is used to assign to a joint a position at the start of the simulation, which will fixed throughout the entire run. In contrast, the Time-Based Dynamic Joint plugin can change the position of the joint at specified time stamps; for example, closing a door after 30 s of simulation time. The Trigger-Based Dynamic Joint plugin can change the joint position from an initial state to an end state if the robot ever gets closer than a specified distance.

Finally, a task specification can be generated using the composable approach based on the scenery models. We opted for generating the task specification in our approach to take advantage of existing mission and task DSLs that meet application and domain-specific requirements for the specification, which would be hard to generalize. As a proof-of-concept, our tool generates navigation tasks tailored to our SUT, but can be easily adapted to generate specifications in other formats. We refer to the tool as the Task Generator, which exploits the geometric information in the FloorPlan model to generate a series of waypoints that form a smaller contour based on the inset of each space in the environment. The tool uses the FloorPlan composable models to extract the free space information, and a configuration file to determine the distance between the room contour and the inset contour. The current prototype generates a list of waypoints using YAML syntax, which is used by the navigation stack.

## 4 Evaluation

To evaluate our approach, we designed three scenarios that demonstrate how to exploit different properties of the scenery for a given test objective. For each scenario we describe the test objective, i.e., the motivation for the test and chosen from the examples in Section 3.1, and the features selected for the test based on the test objective. Note that although testing is context-dependent and the scenarios discussed here take into account a specific System Under Test (SUT), our focus is on *how* to test robot software, not the design or development of a particular navigation algorithm or robot platform. In particular, the goal of the scenarios described in this section is to exemplify how one would use composable scenarios to execute tests to validate the software of a mobile robot. Thus, we mainly focus on the models being used and/or designed as described in Section 3.2.

Our SUT consists of the KELO Robile platform, a mobile robot platform with four active wheels and a 2D laser for navigation. The robot is 0.466 m wide and 0.699 m long. Its software is based on the Robot Operating System (ROS), and uses its navigation stack^6^. This includes the default map_server; move_base to send navigation goals to the robot; the Navfn global planner, the Dynamic Window Approach (DWA) local planner with global and local costmaps; and the Adaptive Monte Carlo Localization (AMCL) algorithm for its pose estimation.

The scenery where the tests are performed is the ground floor of a university building and was modelled using the FloorPlan DSL, as detailed in (Parra et al., 2023). The recreation of the building’s corridors and rooms was achieved by performing measurements of occupancy grid maps captured in the real world. Then the measurements were used to specify the FloorPlan DSL model, and used to generate the occupancy grid and wall mesh.

The test scenarios also exploit the composable models presented in this paper to generate the tasks to be performed and the variations in scenery in which they are executed. First, using the Composable FloorPlan Model, the Task Generator creates task specifications for each room and hallway in the scenarios. Second, we specify an Articulated Scenery Object model that describes the door geometry and joints. Finally, in each scenario we compose this door model with specific scenery instance models into a Gazebo world model that supports the scenario’s testing objective, as will be detailed later.

### 4.1 Scenario 1: functional testing for navigation

#### 4.1.1 Testing objective

The objective of this functional test is to ensure that all components of the navigation stack are correctly integrated and configured^7^. The goal is for the robot to successfully navigate from the starting position to a series of waypoints. In addition, for this paper, we chose to observe the localization component as it is one of the components in the navigation stack that relies on the correct integration with the other components. In this scenario, a successful navigation test means that the robot meets the following functional requirements: (a) reaches all the waypoints, (b) the localization error does not exceed 0.35 m, and (c) the confidence level of the localization component is 95% at minimum.

#### 4.1.2 Models

**FIGURE 4:**
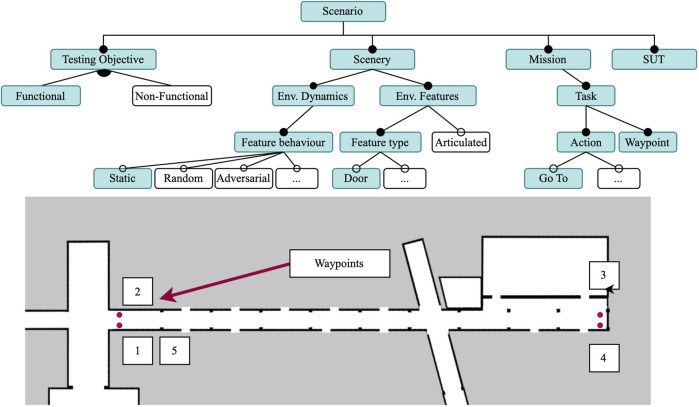
Feature model for Scenario 1, with a static scenery and realistic environmental features (e.g., doors). The task consists of five Go To actions for the four waypoints (red dots) for a mobile robot (SUT).

Given that this is a functional test, we select the features shown in Figure 4 and treat this scenario as a way to obtain a consistent and reproducible baseline. Therefore, the scenery we chose is a static environment with realistic features. The corridor illustrated in Figure 4 is 60 m long, and is part of the FloorPlan model of the university building. It has several features to aid in localization: doorways and doors, columns, and intersections.

The Gazebo world model with static doors was generated by the Scenery Composer using the Composable FloorPlan Model, the articulated scenery door model, and the scenery instance model for the 17 doors. The instance models allow us to specify the initial pose for each door joint, which was set as “closed” (0 rad) for this scenario. Even though the door models are articulated, the doors will remain static throughout the execution.

The navigation task the robot executes was generated by the Task Generator using the Composable FloorPlan model. Its specification consists of a list of waypoints which must be visited in strict order. The generated task specification was manually updated to close the circuit (i.e., five Go To actions in a sequence, including the return to the first waypoint). In this scenario, the waypoints are the four corners of a corridor as shown in Figure 4, and are located at a constant distance of 70 cm from the walls.

#### 4.1.3 Test oracle

A passing test must meet the functional requirements listed in Section 4.1.1. For the experiment, we hold the hypothesis that the robot will be able to localize itself successfully, as the environment is static and has numerous features for correcting the estimation. To measure the localization performance, we rely on two metrics: the error e is computed as the difference between the pose estimation and the ground truth pose $p_{gt}$ , and the standard deviation of the particle cloud, which we use to validate the confidence level. To compute the latter, we obtain the number of times when the difference between the ground truth and the particle cloud is not statistically significant (i.e., not larger than 2σ ). The confidence level is the proportion of those that fulfil Eq. 1,

$${\bar{P}}_{c}-2\sigma \leq p_{gt}\leq {\bar{P}}_{c}+2\sigma$$ (1)

where σ is the standard deviation of the particle cloud, and $\bar{P_{c}}$ is the mean of the particles.

### 4.2 Scenario 2: robustness testing for obstacle avoidance

#### 4.2.1 Test objective

The objective of this test is focused on the robustness of the navigation stack, particularly on the ability of the robot to avoid obstacles in a dynamic environment under stressful conditions. This scenario uses a highly dynamic environment where there is a higher risk of collision with moving doors. The task is now performed in a dynamic version of the scenery, where the doors open and close at random intervals. The challenge for the robot is twofold: first, it has to adapt its plan depending on the status of the doors, which change frequently and randomly. Second, they must avoid colliding with the doors, even if they change state when the robot is very close.

#### 4.2.2 Models

**FIGURE 5:**
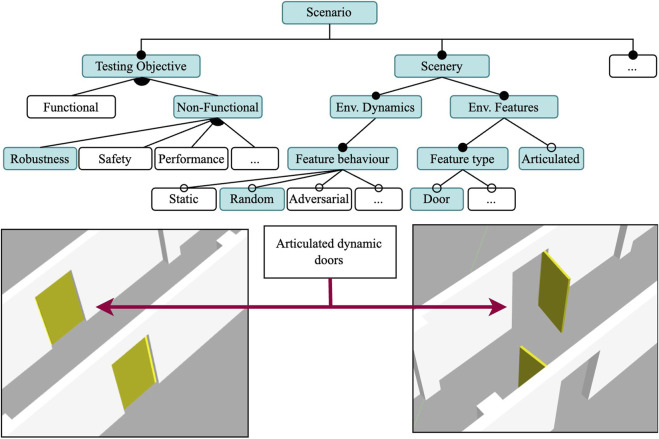
Feature model for Scenario 2: A robustness test in a dynamic environment and randomly actuated doors. The mission and SUT features are the same as in Scenario 1. Door models are articulated.

Using Scenario 1 as a starting point, we increase the complexity to test the robustness of the obstacle avoidance component by making the scenery dynamic, as shown in Figure 5. The scenery for this scenario is mostly the same as the one in Scenario 1, the only difference being the addition of dynamic doors that will open and close at random intervals using the Time-Based Dynamic Joint plugin. Thus, the world file for Scenario 1 is almost identical to the world file for Scenario 2 with the only difference being the use of the plugin at runtime. All positions for all the doors remain the same. Although the task specification is the same, its execution is more complex due to a more challenging environment. When closed, the doors are aligned with the walls of the corridor, keeping the way clear for the robot. When open, the doors are perpendicular to the corridor walls, and partially block the corridor as the doors open towards the inside of the corridor.

The execution models remain mostly the same, the only difference is observed in the world file of the simulation. Dynamic models require a plugin (with its configuration) for them to have a behaviour during the simulation run. For each door, we instantiate a Time-Based Dynamic Joint plugin, which takes as a parameter a JSON file with a sequence of key-frames. The key-frames contain the simulation time at which the joint should move to a particular pose. The doors are closed at 0 rad, and open at 1.7 rad. Each door is independent and has a unique opening/closing sequence, with a randomly assigned state change and simulation time. The sequence for each door remains constant throughout the five runs.

#### 4.2.3 Test oracle

A successful scenario test run is one where the robot successfully avoids all collisions. To measure the effectiveness of the robot to avoid collisions, we look for the smallest distance to an object with respect to the robot’s centre. Given its rectangular shape, a collision occurs when the distance of an object $d_{o}$ to the centre of the robot is $d_{o}\left(x\right)\leq 0.35$ and $d_{o}\left(y\right)\leq 0.2$ . For the simplicity, we discuss $d_{o}$ using the radius of the robot from its corner, i.e., $\sqrt{0.233^{2}+0.35^{2}}=0.42$ although we validate there are no crashes by checking $d_{o}\left(x\right)$ and $d_{o}\left(y\right)$ .

### 4.3 Scenario 3: safety conformance in adversarial environment with task variability

#### 4.3.1 Test objective

Motivated by the near crashes in Scenario 2 (discussed in Sect. 5.2), the objective in this scenario is to validate the conformance of the SUT to one of its safety requirements, namely, that the robot respects the minimum acceptable distance to obstacles and maintains a safety buffer of $d_{s}=0.2$ . More concretely, we validate the ability of the navigation stack to complete a navigation task in an adversarial environment where doors close as the robot approaches them. This means the robot should respond to the environmental changes and conform to its minimum safety distance of 0.2 m, i.e., $min\left(d_{o}\left(x\right)\right)\geq 0.55$ and $min\left(d_{o}\left(y\right)\right)\geq 0.433$ .

In this scenario, we also introduce four variations in the task scale that gradually increase the scenario complexity, creating one sub-scenario for each task. The sub-scenarios were executed 5 times each, which amounts to 20 runs in the simulator for this scenario.

### 4.4 Models

**FIGURE 6:**
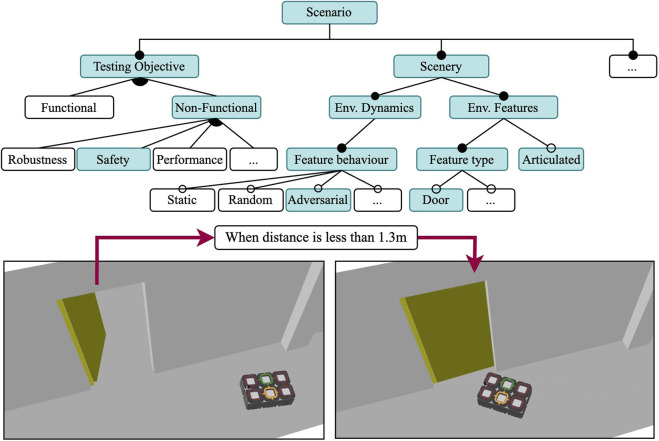
Feature model of Scenario 3 where the scenery door models behave adversarially. Shares the same SUT as Scenario 1 and 2.

To test the safety requirements of the SUT, we select the adversarial behaviour for the dynamic elements of the scenery for this scenario, as can be seen in Figure 6. Following the incremental approach, this scenario will reuse most of the execution models of Scenario 2, but specify an adversarial behaviour for a subset of the doors in the environment. Although we use the same campus re-creation from the previous two environments, our tests are now performed in three rooms rather than one corridor.

**FIGURE 7:**
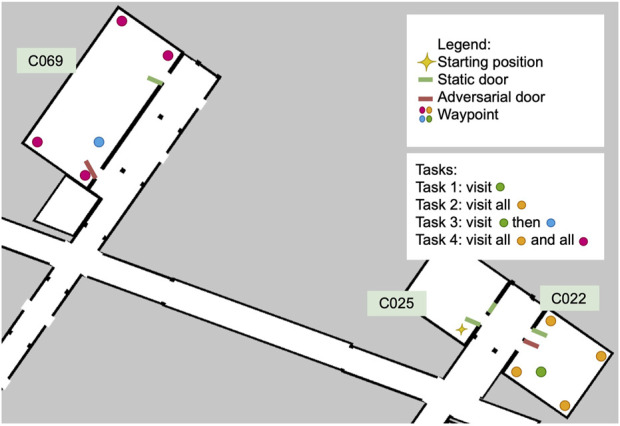
The scenery for Scenario 3, with two adversarial doors (door 17 in C022 and door 12 in C069) and four tasks that vary in scale and number of waypoints.

Instead of random events as in Scenario 2, in this scenery the two dynamic doors are now triggered when the robot is within a distance threshold from the door using the trigger-based dynamic joint plugin. All other doors in the scenery are set to open with the initial joint pose plugin, and remain static throughout the execution. The relevant doors and their behaviour are illustrated in Figure 7. The trigger-based dynamic joint pluginis configured by describing an initial position, a final position, and a minimum distance for the transition trigger. The force and speed of the closing door is not parameterized. When the robot comes to a distance of 1.3 m or closer, an event to close the door triggers. This distance was determined after experimenting with different values, and it ensures that the robot can detect the sudden change in the environment without the door hitting the robot. The order and position of the waypoints was intentionally selected in order to force the robot to plan to pass through the adversarial door.

Because we want the robot to attempt to go through the doorway (as it does not expect the door to close), we chose two different rooms to test this, as shown in Figure 7. In the figure, the four different tasks we composed to gradually increase the complexity of the scenario are also shown. On each task, the number of waypoints to visit and the distances between them increases. The tasks vary in number of waypoints to be visited and distance to the next waypoint. All the tasks start in the same pose in room C025. Task 1 and 2 are relatively short and only involve travelling to C022, while in Tasks 3 and 4 the robot must travel first to C022 and then to C069. Tasks 1 and 3 have a single Go To action in each room for a total of one and two waypoints, respectively; while in Tasks 2 and 4 the robot must perform a total of five and ten Go To actions in sequence, respectively. We name the concrete scenarios to match each task, Scenario 3.1 to 3.4.

#### 4.4.1 Test oracle

We expect the safety requirement of $min\left(d_{o}\left(x\right)\right)\geq 0.55$ and $min\left(d_{o}\left(y\right)\right)\geq 0.433$ to be violated, since the doors will only close when the robot is near the door. However, the expected behaviour is that the robot will avoid collisions in all cases and attempt to move away from the obstacles until it reaches a safe distance again. We observe the changes in speed and angular velocity at the moment the door is triggered, as well as the distance to objects $d_{o}$ , to analyse the robot’s behaviour in context, e.g., whether it is violating the safety distance but moving slowly.

A passing test also requires the robot to complete the tasks successfully. Our hypothesis for this scenario is that the robot will be able to finish the task, but will take more time, as the adversarial elements will just impede the robot to take the shortest possible route. For this comparison, we create two additional sub-scenarios where the doors remain static and which match the most complex tasks at two scales: Task 2 and Task 4, which we name S3.5 and S3.6, respectively.

To measure how well the robot “recovers” once the door closes on its path, we measure the amount of time the robot has obstacles within its safety buffer $t_{d_{s}}$ . We expect the distributions of the total runtime and the total time the Minimum Safety Distance (MSD) $t_{d_{s}}$ was violated for scenarios to behave similarly based on the task scale. Finally, we expect that the behaviour of the robot in an adversarial scenery vs. a static one should not differ substantially other than to avoid the effects of the adversarial door being opened. While we expect $t_{d_{s}}$ to be larger in the adversarial scenery, we expect that the total delay caused by the robot’s reaction to the closing door and the detour caused by the closed door not add more than 30 s for door 17 and 60 s for door 12.

## 5 Results

We ran our experiments in an XMG laptop with 16 GB of RAM and an AMD Ryzen 9 5900HX CPU and running Ubuntu 18.04. The SUT described in Section 4 uses ROS1 noetic. Using the generated artefacts, we execute each scenario 5 times in Gazebo and analyse their results. The models and launch files used to run these scenarios can be found in https://github.com/secorolab/frontiers-replication-package.

### 5.1 Scenario 1

**FIGURE 8:**
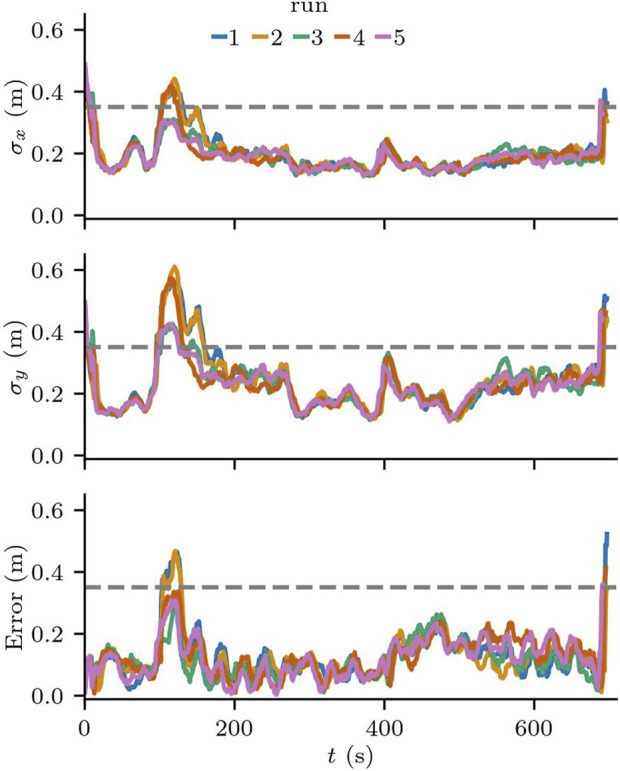
Localization error and standard deviation of the particles along the x and y axis in Scenario 1

In all runs, the robot was able to reach all waypoints and complete the task. The time to complete the task was also consistent, with an average of 696.08 s and a standard deviation of 2.58 s. The behaviour of the robot was consistent across the five runs, with the localization error of 0.1238 m on average, and a maximum value of 0.522 m from run 1. Similarly, the standard deviation of the particle cloud was consistent, as can be seen in Figure 8. As expected, the standard deviation of the particle cloud in y is larger than in x (the direction of travel), and clearly increases whenever the uncertainty about the robot’s orientation increases, i.e., when the robot makes turns. The confidence level of the localization component across all runs was 99.8%.

**FIGURE 9:**
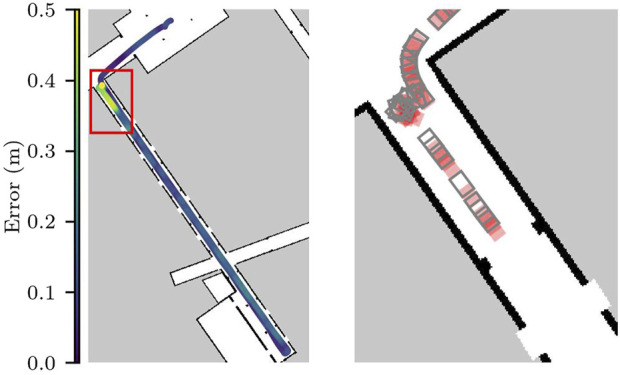
Localization error for run 1 in Scenario 1. On the right the zoomed-in version shows the first 130s of the run.

Despite the error being under the acceptable threshold on average, we can see that the localization requirements are violated briefly when the robot makes turns near the entrance to the hallway. Figure 9 shows the run with the largest error in more detail. On the zoomed in area, we see one of the moments at the beginning of the task, where the localization error and the standard deviation of the particle cloud both reached their maximum values in all runs. This area in particular has a lower number of features for the localization component, as no columns are in range for the laser sensors and there is an intersection right before entering the area.

Although all the runs were completed successfully, only three of the runs met the requirements of the localization component. While the confidence level of the localization component was high, meaning that 99.8% of the time the difference between the pose estimate and the ground truth pose is not statistically significant, the error is larger than the acceptable value for this scenario. This threshold was chosen to guarantee that potential errors in the localization would allow the robot to reach its goals without crashing against the walls.

The results reveal that there are areas in the environment that may require further testing, because although the scenery for this scenario is static and represents the nominal operating conditions for the SUT, the localization component does not meet the application requirements.

### 5.2 Scenario 2

**FIGURE 10:**
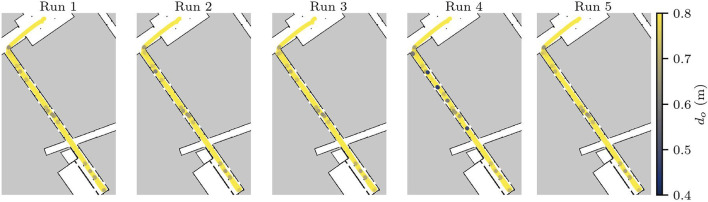
Distance from obstacles in Scenario 2. Distances larger than 0.8 m are shown in yellow, and the darker the purple, the closest the robot was to one of the doors in the hallway.

All test runs for this scenario were successful, as no collisions were detected. The minimum distance to obstacles is shown in Figure 10. One can clearly see that $d_{o}$ decreases near the doorways, as expected. Although the average $d_{o}$ is 1.2 m, run 4 was particularly challenging for the robot; and it is noticeable that in a few locations the dynamic doors almost caused collisions. In this run, on two occasions, $d_{o}$ is less than 5 cm, meaning the robot managed to avoid a collision by merely 3.77 cm and 4.1 cm, respectively.

This scenario builds on top of the static scenery of Scenario 1, and increases the load for the obstacle avoidance component. In a general sense, the dynamic behaviour of the doors in the scenery helps validate the ability of the robot to react to dynamic obstacles in its environment. However, the near misses reveal risks of collision that should be validated against the safety requirements.

### 5.3 Scenarios 3.1–3.6

**FIGURE 11:**
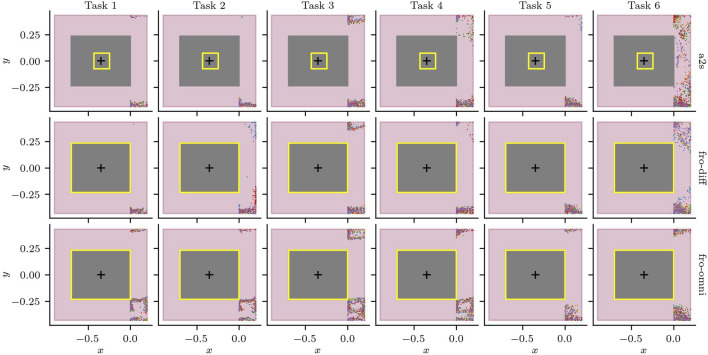
Laser measurements within the safety buffer $d_{s}$ . The robot size (gray), the safety buffer (red) and its configured footprint (yellow).

As a first step to validate the conformance to the safety requirements, we analysed whether there were any obstacles within the 0.2 m safety buffer. To our surprise, we discovered that the SUT struggled with the non-adversarial scenario S3.6, which had one run fail after the robot could not exit C022. Given that the non-adversarial scenery represents the static environment and hence nominal operating conditions, we could immediately conclude that the safety requirements were not being fulfilled. After further inspection, we noticed that the publicly available configuration of the navigation stack^8^ (a2s) had several errors. The robot’s footprint was much smaller and not symmetric around its centre (as can be seen in Figure 11); the laser scan topic used to update the costs for the path planner was using a namespace, i.e., robile_john/scan_front instead of /scan_front; and, finally, although the platform is omnidirectional and the odometry model used by AMCL was configured as such, the path planner was configured to behave as a differential drive robot.

To validate the safety requirements while trying to deliberately provoke a collision (in S3.1–S3.4), we corrected the configuration errors for the differential configuration (fro-diff), and added an omnidirectional configuration (fro-omni). Note that our goal is not to find an optimal configuration, but rather we focus on testing if the new configurations fix the problem we observed. The results of the laser measurements that violate the safety buffer for the five runs for each task and configuration can be seen in Figure 11.^9^

**FIGURE 12:**
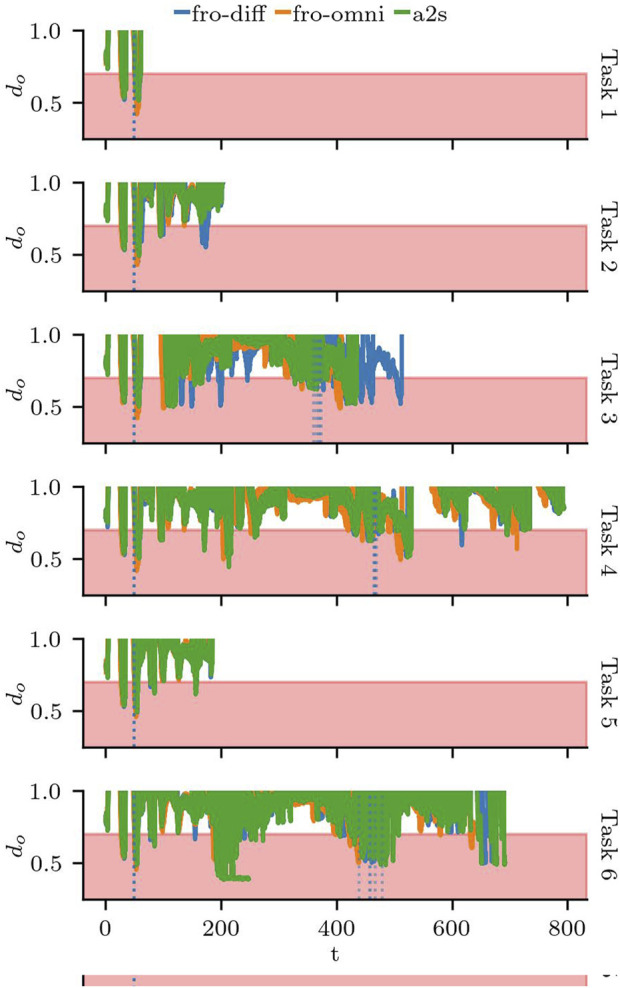
Distance to obstacles $d_{o}$ for Scenario 3. The red area shows the limit at which $d_{s}$ is violated. The dotted lines show when the doors were triggered.

Next, we focus on the behaviour of the robot around the two adversarial doors: door 17 and door 12. We see the moment the doors are triggered as vertical dotted lines in Figure 12. We can see that the behaviour of the robot when door 17 is triggered is consistent regardless of the task. For Scenarios S3.3 and S.6, despite some variation on when door 12 is triggered, the behaviour is similarly consistent. Furthermore, Figure 12 shows that violations to the safety buffer do not only occur with the adversarial doors, but any time the robot passes through or near doorways, and that the combination of the task to execute and the state of the doors contribute significantly to the safety violations.

**FIGURE 13:**
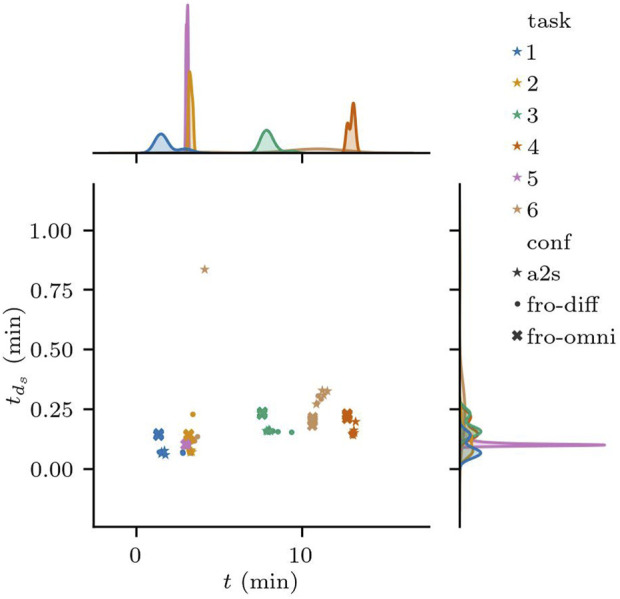
Distribution of the total time the Minimum Safety Distance $t_{d_{s}}$ was violated against the total runtime.

In all the runs, the robot momentarily violates the safety distance on multiple occasions, including the moments where it goes through the non-adversarial doors. Figure 13 shows the distribution for $t_{d_{o}\leq MSD}$ in all the tasks of Scenario 3. Except for the outlier with the larger $t_{d_{s}}$ of the a2s configuration, the difference in the mean of $t_{d_{s}}$ between fro-diff and a2s is not statistically significant. However, both differential configurations had failures on scenario S3.6, while fro-omni was the only configuration to successfully finish all tasks; the trade-off seems to be related to the amount of time the MSD is violated and suggests that there are possible improvements to the latter configuration.

**FIGURE 14:**
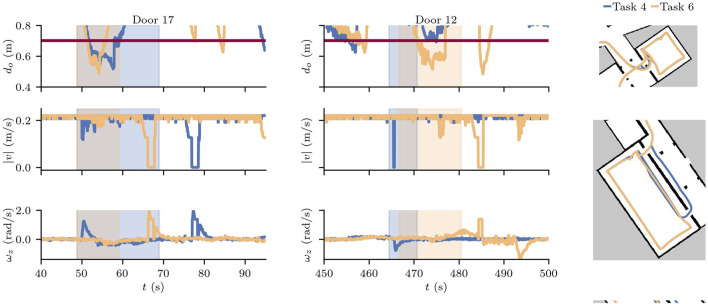
Min. safety dist. for a nominal scenario (Task 6) and a scenario with adversarial doors (Task 4) and configuration a2s.

By comparing runs in sceneries with and without adversarial doors, we can see the effects on the speed when the robot slows down as it attempts to avoid a collision, and the angular velocity changes as it turns to follow an alternate path. Figure 14 shows one run of S3.4 and the same task, but without the effects of the adversarial door in S3.6. We have zoomed in to the two moments where the robot reaches the trigger distance of 1.3 m to door 17 and then door 12.

The total $t_{d_{s}}$ for the different configurations makes the effects of the misconfiguration noticeable. Surprisingly, the original configuration a2s requires more time to complete S3.6 than S3.4, on average 8.9 s more (after excluding the outlier). Surprisingly, the fixed configuration fro-diff also requires more time for S3.6 than S3.4, although it is about half of the misconfigured SUT at 4.8 s. Finally, the fro-omni config behaves as expected, requiring only 1.3 s more for S3.6 than S3.4, which makes these the only successful test runs of scenario 3 in terms of performance.

At a grand scale, these tests reveal that the robot is able to avoid collisions to adversarial obstacles in its environment, attempting to go back to a safe distance as soon as the environmental change is detected. As expected, although the MSD was violated, the robot reacted quickly and the total time MSD was violated did not differ significantly between adversarial and non-adversarial sceneries.

However, upon closer inspection, the test results returned mixed results. Firstly, by using static and dynamic sceneries and a variety of tasks we were able to detect a misconfiguration issue. However, the proposed configurations to fix the issue still do not respect the required safety buffer and need further testing and tuning. Secondly, the tests also revealed that (new) nominal sceneries cause safety violations that still need to be handled, and the effect of the adversarial doors in the performance was overestimated with S3.6 taking longer despite the detour required by the adversarial door in S3.4. Although the scenario tests have met the safety criteria of our oracle regarding $t_{d_{s}}$ , the performance trade-off for the differential configuration was unexpected. Finally, with these examples, we show how the composition plays a key role in the testing process; the interaction between the different features in the scenery and the gradual increase in complexity allowed us to systematically test the SUT and uncover issues that went unnoticed for over a year and in our test runs for S1 and S2.

## 6 Discussion

The results demonstrate that the use of composable and executable scenarios enables the design, specification and execution of tests for a variety of testing objectives. By reusing and composing different scenery models, our approach can gradually increase the complexity of the test scenarios with minimal effort. Due to the context-dependent nature of testing, the results above cannot be generalized to all robots or applications. However, we believe this does not necessarily limit its applicability to other systems due to our focus on the scenery models (which are robot-independent). Furthermore, our evaluation demonstrates how to design, specify and execute test scenarios for systems using the ROS navigation stack (a popular framework used in robotic applications). We now discuss aspects to be considered when applying our approach to a different robot or application.

The transformation and composition process presented in Section 3.3 has a small learning curve. Developers that wish to use our tools, must learn how to specify environments using the FloorPlan DSL and understand the basics of composable models, and making queries on RDF graphs. In its current iteration, because the graph construction relies on the identifiers for each of the elements to be able to successfully compose and query the singular graph, the lack of front-end models for the scenery objects makes this process error-prone. The objects in this paper were limited to doors with hinges, however the composable metamodels for kinematic chains would also support the specification of sliding doors (or objects) by using prismatic joints. The addition of other types of objects is possible, but suffers the same limitations as the current specifications. We hope to extend our DSLs to be able to specify the scenery objects without having to worry about their composable specification.

Although not the focus of this paper, the validation of the scenery and scenario are another area of future development. In (Parra et al., 2023), we presented experiments on the real2sim gap, and shown how developers could validate that the scenery specification reflects a real-world environment. However, we have not yet implemented validation checks after models have been composed into the graph.

The transformation itself is currently handled in two different parts: The Composable FloorPlan model is generated using the textX infrastructure, while other execution artefacts are defined directly in the transformation engine. Ideally, we would like to define these transformations by using transformation rules and a model transformation language, however, target models (e.g., SDF) do not always have publicly available meta-models. Although this could potentially limit the generalization of the approach, the use of templating engines, such as jinja, provides some flexibility and ease of use for extending the type of artefacts supported and customizing the generated model itself. However, we plan to investigate the possibility of using transformation rules for those models with available meta-models to allow for a more systematic transformation.

There are also opportunities for automating the generation pipeline. The process currently is completely under the control of the developers, and each tool is executed manually and independently. On the one hand, this makes the tool modular and allows customizing which aspects of the scenery are to be generated. On the other, it requires additional effort to keep track of and maintain consistency between the models generated by different tools. The modularity of the tools also makes the integration of external or manually-defined models in a scenario possible, at least to some degree. Because composable models can reference other models by their identifiers, those external models can be referenced in the graph and on the templates of the artefacts that use those artefacts (e.g., the 3D Wall mesh or Gazebo models can be referenced by our generated Gazebo world). However, additional effort must be taken to ensure that the IDs, references, and relevant environmental features are valid and consistent with the FloorPlan models.

### 6.1 Related work

In contrast to the autonomous driving domain (Ren et al., 2022), scenario- and simulation-based testing of autonomous mobile robots is desirable; however, this has not been well established (Afzal et al., 2020; Afzal et al., 2021b). In the autonomous driving domain, scenario standards such as ASAM OpenSCENARIO (ASAM, 2022) are emerging to describe common scenery elements, such as roads, streets, traffic signs, and lanes. However, in robotics, environment and scenery modelling is traditionally supported by CAD tools published by multiple vendors. In the context of indoor robotics, and therefore relevant to our work, is the application of these approaches and tools from the architectural domain, where Building Information Modelling (BIM) has been an established technique to model the geometric information of building structural components (e.g., walls, corridors, and windows), as well as semantic hierarchical information (e.g., about the accessibility and connectivity of rooms) (Borrmann et al., 2018). The composable scenario modelling approach introduced in this work targets robotic experts, where BIM is not as prevalent as in other engineering domains. For example, during the interviews, only a single mention of BIM was made. Even though there are numerous 3D software commercially available that implements the BIM standard, modelling scenery is still considered a time-consuming task by robot application developers, as supported by our interviews. In addition, because BIM models support the full building management lifecycle, they introduce many irrelevant dependencies, such as the latest IFC 4.3. x schema^10^ including concepts to define structural building elements (IfcWall, IfcDoor, etc.), but simultaneously introduce concepts for measurements of physical quantities (IfcAbsorbedDoseMeasure, IfcMolecularWeightMeasure, etc.) or building lifecycle management (such as actor roles, including civil engineer or building owner, but also orders, including purchase orders or maintenance work orders). Furthermore, as pointed out by Hendrikx et al. (Hendrikx et al., 2021), BIM cannot be considered accurate or complete for robotic applications.

Another domain related to our approach is the field of computer graphics, specifically procedural content generation approaches, which focus on synthesizing hundreds of environments separated from a single environment description. In robotics, these approaches are typically employed for machine learning applications, as they require a substantial amount of training and testing data that is arduous for manual production. Different approaches use diverse abstractions as inputs, including constraint graphs (Para et al., 2021), handmade drawings (Camozzato et al., 2015), building contours (Lopes et al., 2010; Mirahmadi and Shami, 2012), and natural language descriptions (Chen et al., 2020). A common theme of these approaches is that the output of the generation step is uncontrollable. Input abstractions deliberately exclude spatial relations to keep the input simple because numerous outputs must often conform to the input model. Thus, these spatial relations are synthesized by algorithms and are not controlled by the user. However, not all procedural generational approaches follow this pattern. Some have rich descriptions that allow for a more structured output, while still enabling the generation of variations. For example, the language presented by Leblanc et al. (2011) is an imperative specification language for building indoor environments by performing space operations. These operations involve complex logic to create variation. However, these approaches are not employed in the context of scenario-based testing of robotic systems, where additional models of other agents, dynamic scenery elements, and task specifications need to be composed.

A closely related approach is the Scenic language (Fremont et al., 2019), a probabilistic programming language for generalized environment specification that targets machine-learning applications. Scenic enables the specification of spatial relationships with concrete and logical values. However, Scenic is just a language, and to consume its models, an extra tool is needed to violate our design ambition of having composable scenario models. GzSCenic Afzal et al. (2021a) is a third-party tool that leverages scenic models to generate scenes using the robot simulator Gazebo (Koenig and Howard, 2004). Although these approaches can generate models that are consumable by simulators, they lack the generation of other artefacts for the direct simulation of navigation tasks, such as the occupancy grid map.

Another modelling approach to describe indoor environments is supported by the indoor tagging schema of Open Street Map, which supports modelling a floor plan with tags such as room, area, wall, corridor, and level (floor). The schema was devised for indoor navigation, but can be consumed by any application. Naik et al., (2019) presented an extension to the schema, which was exploited to generate occupancy grid maps and waypoints for navigation. However, these models were not used to generate the simulation models.

### 6.2 Conclusion and future work

In this study, we presented a domain model for features in simulation-based testing scenarios, which we derived from interviews to 14 domain experts. Based on the insights, we propose a composable modelling approach to specify and execute scenarios. Given that the environment representation is one of the challenges mentioned frequently in the interviews, our focus was on facilitating the specification and reuse of scenery models for testing.

The specification of these scenarios starts with a floor plan model that represents the environment in which the robot operates. This specification is done using the FloorPlan DSL from our previous work (Parra et al., 2023). In this paper, we present an extension to the FloorPlan M2M generator, which takes a floor plan model as input and creates a graph representation of its spaces, geometry and elements in JSON-LD, which we call the composable floor plan model. This representation is key to the composability and reusability of the models. Task specifications are generated by our proof-of-concept tool that uses the composable floor plan model to query the geometric information for a target area, and generate waypoints in free space based on its contours. In addition, objects can be composed into the static scenery of the floor plan by specifying an articulated scenery object model (describing the object geometry and its joints), and scenery instance models for each object. These scenery models are also specified in JSON-LD, and are an input for our scenery composer, which traverses the linked graph to generate the required artefacts for the execution of the scenario in simulation. Finally, at runtime, we introduced three Gazebo plugins which set the joint position of the objects composed into the scenery at the start of the simulation, or using time or event-based triggers.

We demonstrated our approach by performing a small simulation-based testing campaign for a mobile robot in a university building. The scenarios gradually increased their complexity, first focusing on validating the navigation stack with functional tests, then performing robustness tests on a highly dynamic environment, and finally, validating the conformance to the safety requirements. The composable aspect allowed us to reuse the static floor plan scenery specified in Parra et al. (2023), and compose static doors for Scenario 1, randomly opening and closing doors in Scenario 2, and doors that would close as the robot approached them in Scenario 3. Surprisingly, only when we ran the third scenario were we able to find a misconfiguration issue in the publicly available navigation stack of our SUT, which had been undetected for over a year despite being in use by multiple groups of students. Normally, this robot operates autonomously within a single room or hallway, and is teleoperated out of the room for the latter, explaining why this issue was not detected until now. This shows that the variation in the scenario features is essential to expose the robot to situations that may generate failures.

Future work includes creating DSLs to specify the scenery objects and instances, and expanding our proof-of-concept task generator to generate task specifications for existing mission and task DSLs. This is a key step to explore the ability of a fully-automated scenario generation approach, which could exploit the Variation DSL introduced in Parra et al. (2023).

## Data Availability

The raw data supporting the conclusions of this article will be made available by the authors, without undue reservation.

## Footnotes

1 The codes and interview fragments are available online at https://github.com/secorolab/floorplan-dsl-interviews.

2
https://gazebosim.org/

3
https://rdflib.readthedocs.io/

4
https://jinja.palletsprojects.com/

5
http://sdformat.org/

6
http://wiki.ros.org/navigation

7 In the scope of this paper, this is a simplified version of an integration test for the navigation stack. As such, we consider it an example of how to validate the functionality of a subsystem within a system-of-systems.

8 This configuration has been used for over a year as part of a robotics course by several groups of students. Available at: https://github.com/a2s-institute/robile_navigation/tree/noetic/config

9 We also ran the tests for S1 and S2 with the new configurations, and validated that they perform similar to the original a2s configuration.

10
https://github.com/buildingSMART/IFC4.3.x-output/blob/master/IFC.xsd
