# Supplementary material for: Composable and executable scenarios for simulation-based testing of mobile robots
Source: Front Robot AI. 2024 Aug 2;11:1363281. doi: 10.3389/frobt.2024.1363281 (PMC11327003; doi:10.3389/frobt.2024.1363281)
Supplement: Supplementary file 1 [file DataSheet1.pdf]

frontiers

Supplementary Material

1 SUPPLEMENTARY TABLES AND FIGURES

Table S1. List of interviewees. Reproduced from Parra et al. (2023)

Id.	Academic background	Degree	Field	Experience
P1	Mechatronics Engineering	M.Sc	Industry/Academia	8
P2	Mechatronics	M.Sc	Industry	7
P3	Robotics and Automation	M.Sc	Industry	5
P4	Computer Science	M.Sc	Industry	4
P5	Electronics & Communication	B.Sc	Academia	3
P6	Computer Science	M.Sc	Industry/Academia	8
P7	Industrial Engineering	PhD	Academia	15
P8	Mechatronics	M.Sc	Academia	7
P9	Mechatronics	M.Sc	Academia	6
P10	Mechatronics Engineering	M.Sc	Industry	10
P11	Computer Science	M.Sc	Academia	8
P12	Computer Science	M.Sc	Industry	15
P13	Electronics & Communication	M.Sc	Academia	5
P14	Control Engineering	PhD	Industry/Academia	11

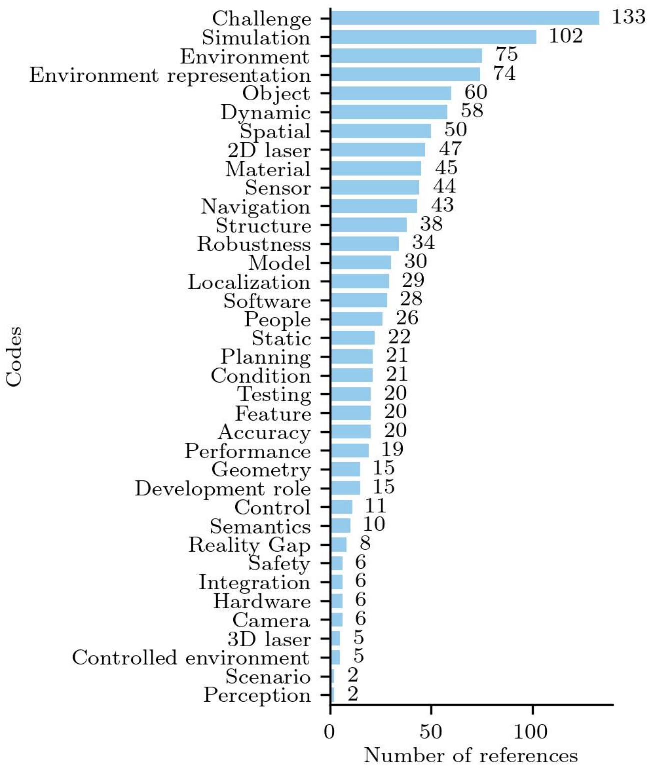

Figure S1. Distribution of the number of references per code in participant interviews

REFERENCES

Parra, S., Ortega, A., Schneider, S., and Hochgeschwender, N. (2023). A thousand worlds: Scenery specification and generation for simulation-based testing of mobile robot navigation stacks. In *IEEE/RSJ Intl. Conf. on Intell. Robots and Syst. (IROS)*. 5537–5544. doi:10.1109/IROS55552.2023.10342315
